# Alternative new mesenchymal stem cell source exerts tumor tropism through ALCAM and N-cadherin via regulation of microRNA-192 and -218

**DOI:** 10.1007/s11010-016-2909-5

**Published:** 2016-12-30

**Authors:** Ran Kim, Sang In Park, Chang Youn Lee, Jihyun Lee, Pilseog Kim, Sekyung Oh, Hojin Lee, Min Young Lee, Jongmin Kim, Yong-An Chung, Ki-Chul Hwang, Lee-So Maeng, Woochul Chang

**Affiliations:** 1 Department of Biology Education, College of Education, Pusan National University, Pusan, 609-735 South Korea; 2 Institute of Catholic Integrative Medicine, Incheon St. Mary’s Hospital, The Catholic University of Korea, College of Medicine, Incheon, 403-720 South Korea; 3 Brain Korea 21 PLUS Project for Medical Science, Yonsei University College of Medicine, Seoul, 120-752 South Korea; 4 Department of Neurology and Neurological Sciences, Stanford University School of Medicine, Stanford, CA 94305 USA; 5 Department of Pharmacology, Yale University School of Medicine, New Haven, CT 06520 USA; 6 Department of Molecular Physiology, College of Pharmacy, Kyungpook National University, Daegu, 702-701 South Korea; 7 Department of Life Systems, Sookmyung Women’s University, 52 Hyochangwon-gil, Seoul, 140-742 South Korea; 8 Institute for Bio-Medical Convergence, College of Medicine, Catholic Kwandong University, Gangwon-do, 210-701 South Korea; 9 Catholic Kwandong University International, St. Mary’s Hospital, Incheon, 404-834 South Korea

**Keywords:** Synovial fluid derived-mesenchymal stem cells, Tumor tropism, Activated lymphocyte cell adhesion molecule, N-Cadherin, microRNA-192, microRNA-218

## Abstract

Gliomas are the most common type of malignant primary brain tumors. Some treatments of gliomas exist, but they are rarely curative. Mesenchymal stem cells (MSCs) are emerging as potential modes of targeted cancer therapy owing to their capacity for homing toward tumor sites. It has been proposed that MSCs derived from various sources, such as bone marrow, adipose tissue and umbilical cord blood, can be used as cell-based therapy for brain tumors. Here, MSCs obtained from the synovial fluid of osteoarthritis or rheumatoid arthritis patients were investigated as therapeutic candidates. Specifically, we compared migratory and adhesive abilities, as well as expression levels of related genes and microRNA in bone marrow derived-MSCs (BMMSCs), adipose derived-MSCs (ADMSCs), and synovial fluid derived-MSCs (SFMSCs) after treatment with conditioned medium from gliomas. Migration and adhesion of SFMSCs increased through upregulation of the activated lymphocyte cell adhesion molecule (ALCAM) and N-cadherin by microRNA-192 and -218 downregulation, similar to BMMSCs and ADMSCs. Migratory capacities of all types of MSCs were evaluated in vivo, and SFMSCs migrated intensively toward gliomas. These results suggest that SFMSCs have potential for use in cell-based antitumor therapies.

## Introduction

Malignant gliomas are the most prevalent type of primary brain tumors affecting the central nervous system [1, 2]. Despite recent technological advances in surgical resection, such as complementation with radiotherapy and chemotherapy, such treatment is rarely curative in cases of extremely poor prognosis [3, 4]. This difficulty is a result of the highly invasive and infiltrative growth patterns of gliomas, which commonly diffuse into surrounding brain tissues [1]. In addition, the aforementioned treatments have several limitations. For example, surgical resection using currently available technologies cannot eliminate all tumor cells [5]. Radiotherapy generally results in irradiation of normal brain tissues as well as the target tumor, causing multiple side effects [1]. Chemotherapy also has limited effects because most chemicals have difficulty crossing the blood brain barrier [6]. Recent studies have proposed using tumor tropism of mesenchymal stem cells (MSCs) to overcome the limitations of these approaches.

MSCs are stem cells with self-renewal capacity and multipotentiality that represent an alternative source of therapeutic stem cells [7, 8]. In recent years, MSCs have received a great deal of attention owing to their ability to target tumors, including gliomas, making them a promising candidate for cell-based therapy [9]. Previous studies have demonstrated that bone marrow-derived MSCs (BMMSCs) and adipose tissue-derived MSCs (ADMSCs) showed tumor-tropic migratory capacities and therapeutic efficacy against gliomas [4, 10]. However, no studies have investigated the use of synovial fluid-derived MSCs (SFMSCs) from osteoarthritis (OA) or rheumatoid arthritis (RA) patients. Additionally, further studies are needed to understand the mechanisms that regulate the migratory and adhesive abilities of MSCs to improve their application as therapeutic antitumor vehicles.

MSCs can express migratory and adhesive related molecules in response to tumor cell-derived substances and factors that can specifically attract stem cells [1]. Activated lymphocyte cell adhesion molecule (ALCAM) is a member of the cell adhesion molecule (CAM) family that acts as a homophilic adhesion protein to induce heterophilic binding to CD6 in hematopoiesis [11]. N-cadherin, one of the classic cadherins, is also related to changes in cell motion, migration, invasion, and adhesion [12]. MicroRNA (miR) is small, non-coding single stranded RNA that plays a role as a novel class of gene regulators by promoting degradation of mRNA transcripts and down-regulating protein translation [13]. MiR-192 and -218 are candidate regulators for modulation of migratory and adhesive abilities of MSCs via changes in ALCAM and N-cadherin expression, respectively. mRNA encoding the adhesion molecule ALCAM is targeted by miR-192 and downregulated at the mRNA and protein levels [14]. Moreover, previous study reported suppression of cell migratory ability indicates that N-cadherin is a direct target gene of miR-218 [15]. Regulation of key proteins associated with cell behaviors and regulatory miR is important because of the potential for novel strategies for treatment of cancer.

In this study, we investigated migratory, adhesive and spreadable abilities of BMMSCs, ADMSCs, and SFMSCs cultured using conditioned media (CM) from gliomas. The expression level of ALCAM and N-cadherin, as well as their regulators miR-192 and -218, were measured in three types of MSCs using real-time quantitative RT-PCR (qRT-PCR). In addition, each lineage of MSCs was injected to check its capacity to migrate toward tumor sites in vivo. The results of this study indicate that SFMSCs have potential for use as a new therapeutic vehicle for cell-based anticancer therapies based on comparison to the efficiency of other MSCs lineages.

## Materials and methods

### Isolation of SFMSCs from patients with osteoarthritis

SFMSCs were prepared as previously described [16]. Briefly, synovial fluid (SF) samples were obtained from OA patients by joint puncture. The samples were then washed with phosphate buffered saline (PBS, Hyclone, USA) containing 0.1 M EDTA for 10 min at 363×*g*. Next, cells were collected and suspended in Dulbecco’s modified Eagle’s medium (DMEM, Hyclone) containing 10% fetal bovine serum (FBS, Invitrogen, USA) and incubated at 37 °C under 5% CO_2_. After 3 days, the medium was replaced to remove non-adherent cells. Ethical approval for the use of SFMSCs was obtained from the Institutional Review Board of Catholic University Medical center.

### Cultures of BMMSCs, ADMSCs, and SFMSCs

Three types of MSCs were expanded as previously reported [16, 17]. BMMSCs (Cat no. PT-2501, Lonza, USA) were maintained in DMEM containing 10% FBS and 1% antibiotic-penicillin/streptomycin solution. ADMSCs (Cat no. R7788-115, Invitrogen) were expanded using MesenPRO RS™ basal medium, growth supplements and 2% FBS. SFMSCs were plated in complete culture medium consisting of α -MEM (Invitrogen) with 10% FBS, 1% antibiotic-penicillin/streptomycin solution and 250 ng/ml amphotericin B (Invitrogen). All types of cells were incubated at 37 °C under a humidified atmosphere containing 5% CO_2_.

### Preparation of conditioned glioma media

Human glioma cell lines were obtained from the American Type Culture Collection (USA), after which primary cells were obtained from fresh specimens of glioma-bearing patients undergoing surgery following ethical approval and written informed consent. U87 cell line which is a human primary glioblastoma cell line is commonly used as experimental models of glioblastoma multiforme [37]. GM16 is human gingival keratinocytes that are immortalized by human papillomavirus (HPV) type 16 E6/E7 oncogenes [38]. Gliomas that were 70–80% confluent were re-fed with serum-free medium (SFM) containing antibiotic–antimycotic. The cultured CM was collected after 48 h of incubation and stored at 4 °C or −80 °C before use in the subsequent experiments.

### Cell migration assay

Three types of MSCs were examined by modified Boyden chamber assay using transwell plates with 8 μm pore filters (Corning, USA). The cells were seeded into the upper chambers, after which gliomas-CM was added to the lower chambers. Following incubation for 6 h, cells that had not migrated through the upper side of the filters were scraped off with swabs. The filters were then stained with hematoxylin, after which cells that had migrated to the lower side were counted using an inverted microscope (Nikon Co., Japan). The migration assay was conducted in triplicate.

### Analysis of cell adhesion and spreading

Adhesion and spreading assays were performed as previously described [18]. To determine the adhesion of each type of MSC, 2 × 10^4^ cells were added to 6-well plates (Corning) and incubated for 1 h with CM from gliomas. Cells were carefully washed with PBS, then fixed in 3% paraformaldehyde. Photographs of a minimum of four fields were taken of each well, after which cells were counted using the Meta-Morph imaging software, version 7.5 (Molecular Devices, USA). For spreading analysis, three types of MSCs were incubated for 4 h in 2-well plates (Corning). Samples were then stained with Coomassie blue (Santa Cruz Biotechnology, Inc., USA) and counted using a phase contrast microscope. Each experiment was repeated three times.

### Real-time quantitative RT-PCR

Total RNA was extracted from gliomas CM-treated BMMSCs, ADMSCs, and SFMSCs using an RNeasy mini kit (Qiagen, USA). Next, 1000 ng of total RNA was transcribed with an Omniscript RT kit (Qiagen) and amplified in triplicate using a MyiQ Single-Color RT-PCR Detection System (TaKaRa, Japan). The following primer sequences were used: ALCAM, forward 5′-ACTGGCAGTGGAAGCGTCAT-3′ and reverse 5′-CAGCAAGGAGGAGACCA-3′; N-cadherin, forward 5′-CACTGCTCAGGACCCAGAT-3′ and reverse 5′-TAAGCCGAGTGATGGTCC-3′. The relative expression of each target gene was calculated using the threshold cycle (ΔCt) method after normalizing the ΔCt to the cycle number of GAPDH.

### Luciferase assay

The target miR was selected using a public database (Target Scan; www.targetscan.org). Molecular beacons (Bionics Co., Korea) were designed to detect the expression of miR in single cells: miR-192-5p, 5′-CUGACCUAUGAAUUGACAGCC-3′, miR-218-5p, 5′-UUGUGCUUGAUCUAACCAUGU-3′. rMSCs were plated at 2.5 × 10^4^ cells/well in 24-well plates (Corning, USA). After 48 h, pmirGLO vectors containing the ALCAM and N-cadherin binding sites for miR-192 and -218 were co-transfected with miR-192 and -218 or the negative control using Lipofectamine 2000 (Invitrogen), respectively. Renilla luciferase was used to normalize cell numbers and transfection efficiency. After an additional 48 h, luciferase activity was measured using the Dual Luciferase assay (Promega CO., USA) according to the manufacturer’s instructions. Each assay was repeated three times.

### Animals and brain tumor model

Male athymic nude mice (6–8 week old; Charles River Laboratories) were used in accordance with the guidelines of the Institutional Animal Care and Use Committee of the Incheon Catholic University Medical School. Brain tumor models were generated as previously described [19]. For the intracranial xenografts of human gliomas, animals were anesthetized using an intraperitoneal injection of ketamine/xylazine and inoculated with 1 × 10^5^ U87-Luc cells in 3 µl PBS into the right frontal lobe.

### In vivo migration assay

After 7 days of tumor cell inoculation, NEO-LIVETM-Magnoxide675-labeled MSCs were injected into the tail vein. Migration of three types of MSCs toward the tumor was then assessed by direct visualization at 4 h and 1, 5 and 8 days using the Maestro in vivo imaging system. The fLuc substrate D-luciferin (150 mg luciferin/kg body weight), which was used to measure tumor growth, was delivered by intraperitoneal injection.

### Statistical analysis

All data are expressed as the mean ± SE. Statistical differences between two different test conditions were estimated by a Student’s *t* test. Comparison of more than two groups was accomplished using one-way ANOVA with the Bonferroni test. A P < 0.05 was considered statistically significant.

## Results

### Conditioned media of glioma enhances migratory ability of SFMSCs

**Fig. 1:**
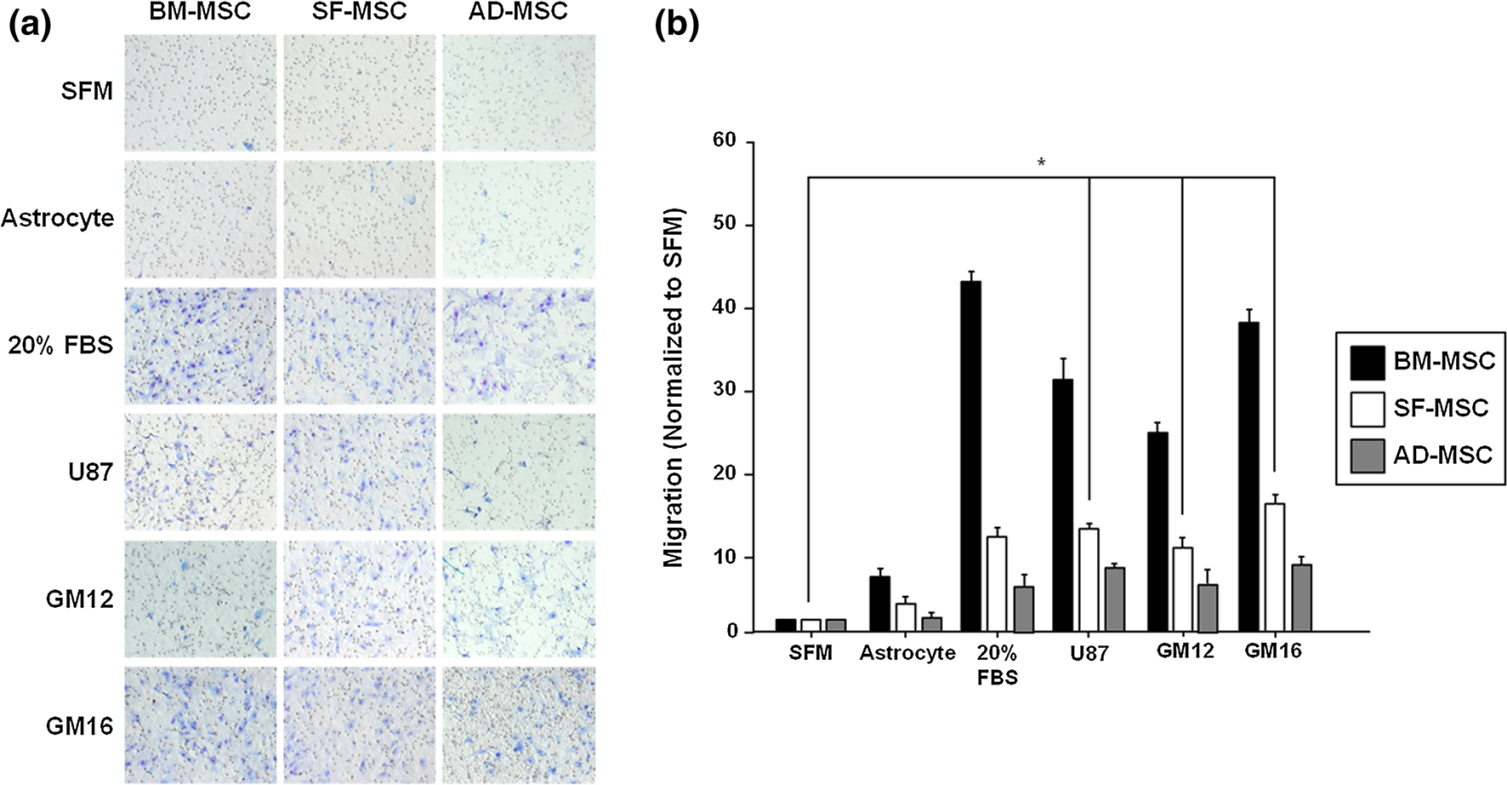
Migratory ability of gliomas-CM treated SFMSCs in vitro. **a** Three types of MSCs showed changing migratory abilities in response to each CM. **b** Migratory abilities of SFMSCs treated with gliomas-CM were normalized and significantly enhanced relative to SFM. Data are expressed as the mean ± SD, *P < 0.05

Tumor homing of BMMSCs, ADMSCs, and SFMSCs by glioma-secreted factors was examined and compared using a transwell assay. SFM and CM of astrocytes from normal brain tissues were used as negative controls and 20% FBS DMEM medium was used as a positive control. The microphotographs showed that cell migration of the three types of MSCs was enhanced relative to the negative controls (Fig. 1a). All types of MSCs migrated in the presence of SFM, and CM from astrocytes cultivation was low, but migration increased in the in the presence of 20% FBS DMEM. Treatment of CM with a commercial cell line of gliomas (U87) and those derived from patients (GM12 and GM16) significantly increased cell migration of all types of MSCs. The relative migration of SFMSCs increased 12.0-fold in the U87 cells, 9.2-fold in the GM12 cells, and 15.5-fold in the GM16 cells relative to SFM (Fig. 1b). These findings indicate that treatment with glioma-derived CM can enhance migration of MSCs derived from OA patients’ SF in response to glioma-secreted factors.

### Conditioned glioma media enhances adhesive and spreading abilities of SFMSCs

**Fig. 2:**
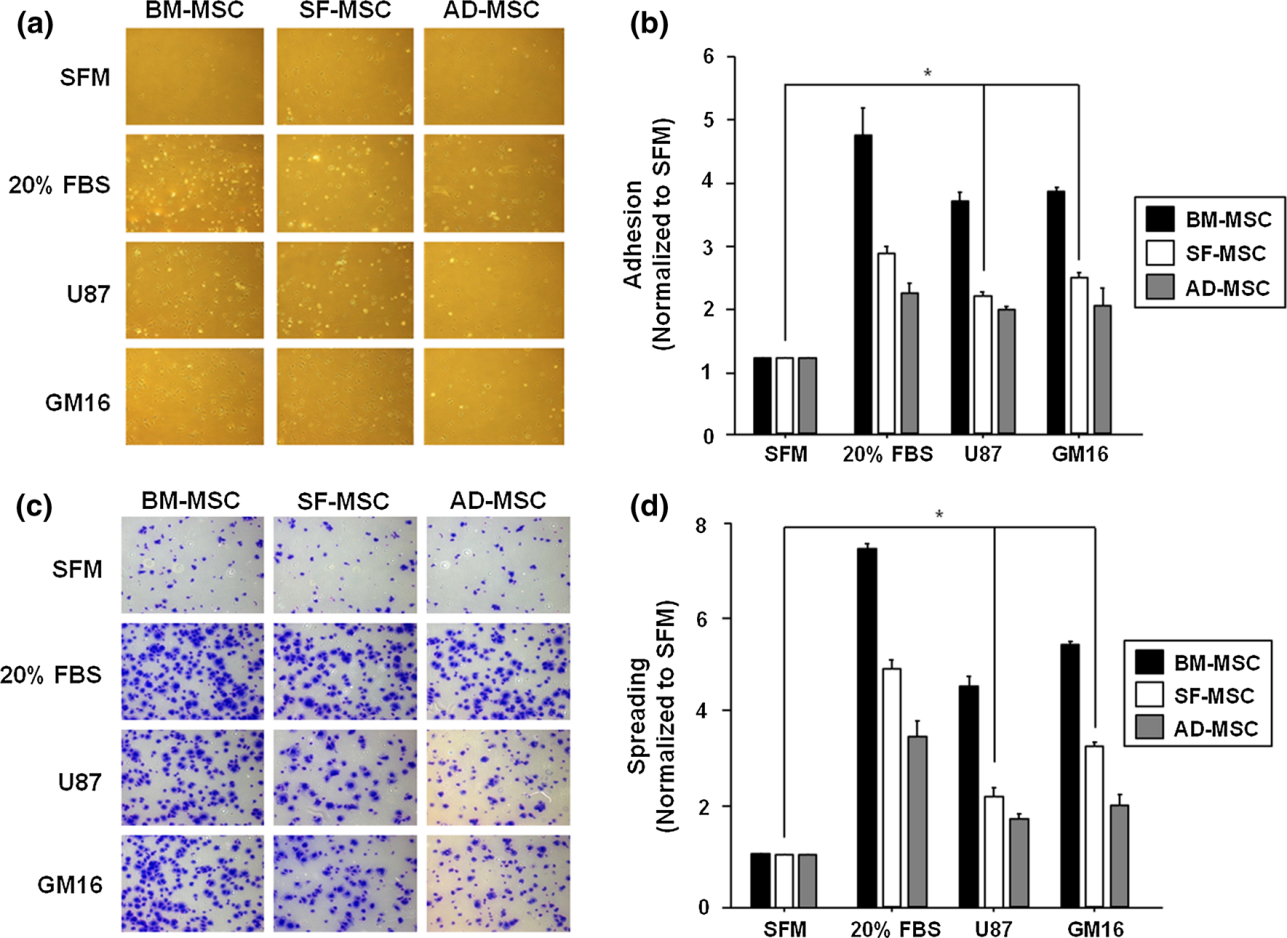
Adhesion and spreading of gliomas-CM treated SFMSCs in vitro. **a** Adhesive abilities of the three types of MSCs were evaluated after treatment of each CM. **b** SFMSCs significantly enhanced the adhesion capacity compared to treatment with SFM. **c** The spreadable abilities of all types of MSCs treated with each CM were confirmed. **d** Spreadable abilities of SFMSCs were increased, indicating a similar pattern as to the adhesion assay. Data are expressed as the mean ± SD, *P < 0.05

The potential of CM from gliomas was determined to compare its influence on the abilities of BMMSCs, ADMSCs, and SFMSCs to adhere and spread in vitro. Microscopy revealed that all types of MSCs had enhanced adhesion capacity and spreading when grown in CM relative to SFM (Fig. 2a, c). Adhesion of SFMSCs was 2.0-fold and 2.4-fold higher in U87-CM and GM16-CM, respectively (Fig. 2b). The ability of SFMSCs to spread was significantly increased by 2.4-fold in U87-CM and 3.8-fold in GM16-CM, which was similar to the results of the adhesion assay (Fig. 2d). Secreted factor in gliomas-CM may be responsible for the increased adhesion and spreadability of MSCs derived from OA patients’ SF.

### Upregulation of migration- and adhesion-related molecules

**Fig. 3:**
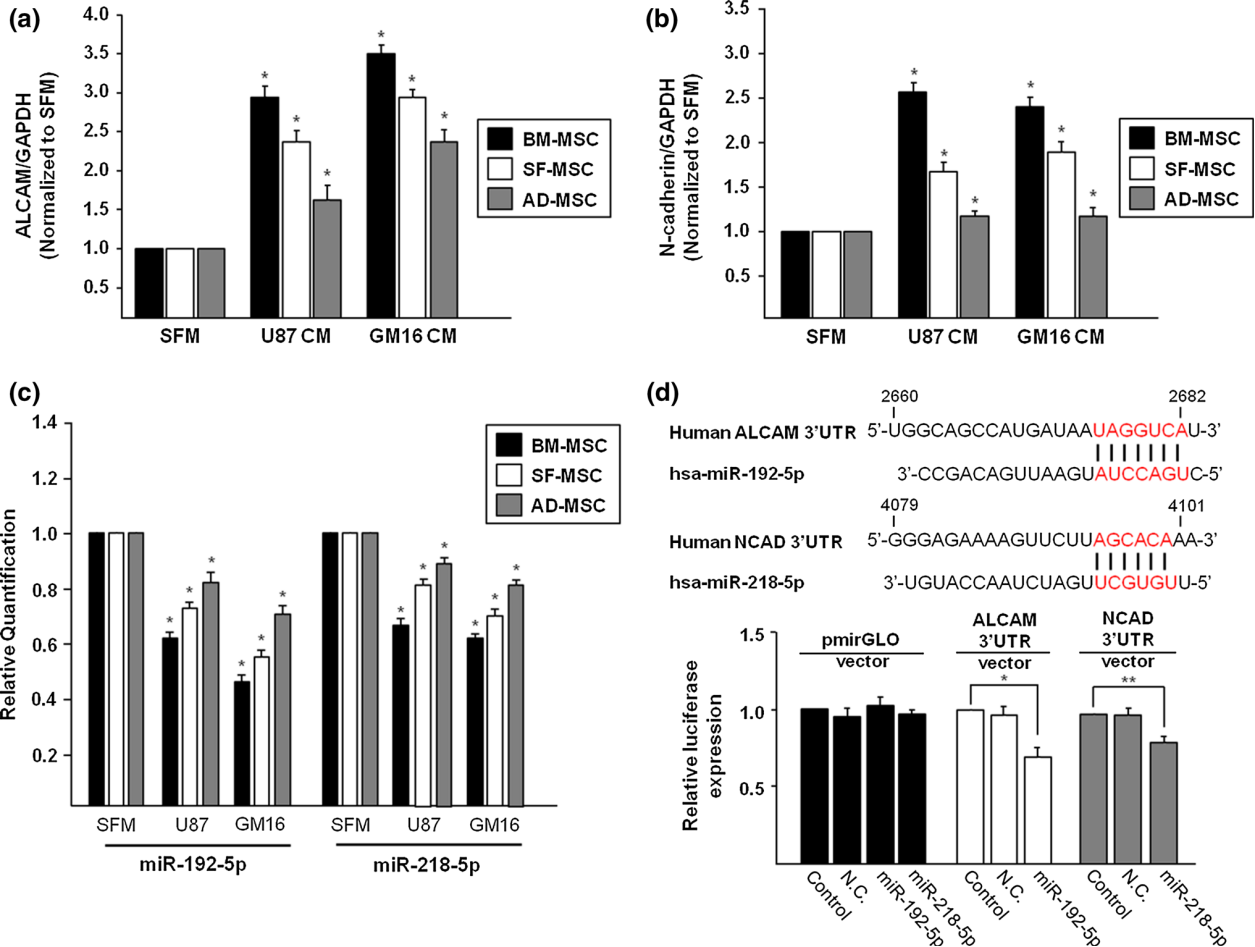
MiR-192 and -218 mediated expression of ALCAM and N-cadherin on SFMSCs treated with gliomas-CM. **a** mRNA expression of ALCAM in SFMSCs was greater than that in SFM following treatment with glioma-CM. **b** N-cadherin expression was significantly upregulated in SFMSCs with CM from two gliomas. **c** Expression of miR-192 and -218 was downregulated in SFMSCs by gliomas-CM treatment. **d** The sequences of miR-192 and -218 showed that they can bind to regulate expression of target genes. Luciferase assay revealed that each target gene was regulated by miR-192 and -218, respectively. Data are expressed as the mean ± SD, *P < 0.05

The mRNA expression of ALCAM and N-cadherin, which play critical roles in the regulation of cell migration and adhesion, was quantified by qRT-PCR. The expression of ALCAM in the three types of MSCs following treatment with conditioned medium of U87 and GM16 was higher than that of the control. mRNA expression of ALCAM in SFMSCs in SFMSCs increased by more than 2.4- and 2.9-fold in response to treatment with CM from U87 and GM16, respectively (Fig. 3a). N-cadherin was also highly expressed in all types of MSCs in the same pattern as that of ALCAM. The level of expression of the N-cadherin gene was significantly upregulated in SFMSCs with CM from two gliomas by 1.7- and 1.9-fold, respectively (Fig. 3b). These migration- and adhesion-related genes may enhance migration of MSCs toward and adhesion of MSCs at the tumor area.

### ALCAM and N-cadherin are the predicted targets of miRNA-192-5p and miRNA-218-5p

We focused on potential miR targeting ACALM and N-cadherin. Based on Target Scan, miR-192-5p and miR-218-5p were selected as negative regulators of the related cell migration and adhesion genes. Both miR-192-5p and miR-218-5p were expressed in significantly lower levels in all types of MSCs relative to the control. Moreover, treatment with CM from both gliomas decreased expression of miR-192-5p and miRNA-218-5p in all types of MSCs (Fig. 3c). Finally, targeting the 3′-UTR of ALCAM and N-cadherin resulted in decreased luciferase activity (Fig. 3d) by miR-192 and -218, respectively.

### Comparison of in vivo migratory capacity of MSCs derived from different sources

**Fig. 4:**
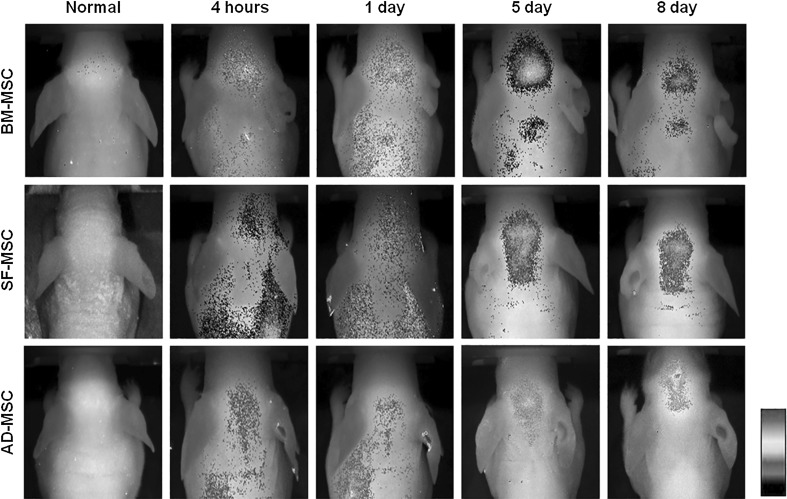
In vivo migration assay in mouse brain tumor models. Migratory capacities of BMMSCs, ADMSCs, and SFMSCs toward glioma in vivo showed SFMSCs have satisfactory tumor homing

To compare the migratory capacities of BMMSCs, ADMSCs, and SFMSCs toward glioma in vivo, three types of MSCs were injected into the tail vein following transplantation of U87 glioma cells into the brains of mice. All types of fluorescence labeled MSCs were detected in the tumor area. BMMSCs migrated most intensively from the initial injection site to the tumor area, and migration capacity of SFMSCs toward tumors was much greater than that of ADMSCs (Fig. 4).

## Discussion

Tumor tropism is a complex, multistep process used by many types of cells to migrate from a distant location to a tumor site [9]. MSCs also have the tropism based on their inherent ability to migrate to sites of tissue injury or damage [10, 20]. Theory of MSCs homing to injured or inflamed tissues is supported much evidence. Transplanted MSCs travel in the blood stream, undergo transendothelial migration through vascular walls and invade into the target tissues [31]. Tumor-tropic migratory capacity is mediated by tumor cells or the surrounding tissue [4, 23]. MSCs homing is triggered by tumor-cell specific receptors and soluble tumor derived factors [9]. The release of chemotactic gradients from tumors causes specific migration to gliomas, inducing MSCs to migrate and modulate the tumor microenvironment [24, 25]. Upon arrival in the tumor, MSCs can show antitumor properties, and can decrease cancer cell growth [21, 22]. Accordingly, MSCs have received great attention as potential vehicles for delivery of various therapeutic drugs or genes directly to tumor cells [2].

This study was conducted to identify a new source of MSCs as a tumor-tropic vehicle. Many studies have shown that some MSCs lineages derived from different sources, including bone marrow, adipose tissue, and umbilical cord blood, have the ability to migrate to tumors in vitro and in vivo [4, 10]. However, there have been no studies conducted using MSCs derived from synovial fluid, despite their potential for use as a vehicle for gliomas therapy. Recently, SFMSCs have attracted attention owing to the developmental plasticity and therapeutic potential of stromal cells isolated from synovial fluid. Their multipotency and ex vivo proliferation capacity are advantages for using cell based-therapies [34, 35]. And SFMSCs produce immunomodulatory molecule, human leukocyte antigen-G (HLA-G) which show immunosuppressive effect on activated T cells [36]. These cells can be also harvested easily during arthrocentesis or routine arthroscopic examination of patients with OA or RA without harming normal tissue [26, 27]. SFMSCs which we used in the present paper were already characterized and confirmed using method of our previous study [28]. In addition, SFMSCs from patients could be cultured for at least seven passages without altering their morphology and proliferating capacity relative to other MSCs [28]. SFMSCs are commonly used for treatment of OA and RA [16, 29, 30]. In this study, we focused on their potential for use as a novel cell source for cancer therapy, as well as their merits and characteristics.

The results of this study demonstrate that SFMSCs have potential tumor homing capacity along with BMMSCs and ADMSCs. To confirm the migratory and adhesive abilities of SFMSCs in response to CM from gliomas, in vitro assays were performed to compare BMMSCs, ADMSCs, and SFMSCs. The results indicated that SFMSCs can migrate toward gliomas and adhere and spread on tumor sites effectively (Figs. 1, 2). Although the mechanisms for the migration and adhesion of MSCs are not yet fully understood, we speculated that the expression of related molecules would change in MSCs. To test, this, we evaluated ALCAM and N-cadherin, which play critical roles in the regulation of cell migration and adhesion. The expression of these genes was higher in the tested cells than in the control. Next, we investigated the miR candidates, miR-192 and -218, which regulate migration and adhesion molecules of MSCs. Our data showed that miR-192 and -218 were downregulated in all types of MSCs in response to gliomas-CM, including those of SFMSCs (Fig. 3). After in vitro experiments, the migratory capacities of three types of fluorescent labeled MSCs were compared in vivo (Fig. 4).

## Conclusion

In conclusion, we suggest a new therapeutic vehicle cell source with satisfactory tropism property for tumor therapies. SFMSCs are a promising cancer targeted-delivery vehicle by loading interleukins, interferons, pro-apoptotic proteins, oncolytic viruses, antiangiogenic agents, nanoparticles with anticancer drug, prodrugs [32, 33]. Although SFMSCs have therapeutic potential to treat malignant gliomas, the effects using SFMSCs have not been confirmed until now. Cancers occurred in other organs as well as brain tumors may be applied and showed improvement by utilization of SFMSCs as cell-based antitumor therapies.
